# The Effect of Rare-Earth Elements on the Morphological Aspect of Borate and Electrocatalytic Sensing of Biological Compounds

**DOI:** 10.3390/bios13100901

**Published:** 2023-09-22

**Authors:** Roman Morozov, Dalibor Stanković, Viacheslav Avdin, Dmitri Zherebtsov, Mikhail Romashov, Anastasia Selezneva, Daniil Uchaev, Anatoly Senin, Alexander Chernukha

**Affiliations:** 1 Nanotechnology Research Center, South Ural State University, 454080 Chelyabinsk, Russia; morozovrs@susu.ru (R.M.); avdinvv@susu.ru (V.A.); zherebtcovda@susu.ac.ru (D.Z.); romashovms@susu.ru (M.R.); hardcorestacy6@gmail.com (A.S.); uchaevda@susu.ru (D.U.); seninav@susu.ru (A.S.); chernukhaas@susu.ru (A.C.); 2 Faculty of Chemistry, University of Belgrade, 11000 Belgrade, Serbia

**Keywords:** rare earth, borates, pyridoxine, electrochemical detection, B6

## Abstract

Adjusting the morphological characteristics of a material can result in improved electrocatalytic capabilities of the material itself. An example of this is the introduction of rare-earth elements into the borate structure, which gives a new perspective on the possibilities of this type of material in the field of (bio)sensing. In this paper, we present the preparation of borates including La, Nd and Dy and their application for the modification of a glassy carbon electrode, which is used for the non-enzymatic detection of a biologically relevant molecule, vitamin B6 (pyridoxine). Compared with the others, dysprosium borate has the best electrocatalytic performance, showing the highest current and the lowest impedance, respectively, as determined using cyclic voltammetry and impedance tests. Quantitative testing of B6 was performed in DPV mode in a Britton–Robinson buffer solution with a pH of 6 and an oxidation potential of about +0.8 V. The calibration graph for the evaluation of B6 has a linear range from 1 to 100 μM, with a correlation coefficient of 0.9985 and a detection limit of 0.051 μM. The DyBO_3_-modified electrode can be used repeatedly, retaining more than 90% of the initial signal level after six cycles. The satisfactory selectivity offered a potential practical application of the chosen method for the monitoring of pyridoxine in artificially prepared biological fluids with acceptable recovery. In light of all the obtained results, this paper shows an important approach for the successful design of electrocatalysts with tuned architecture and opens new strategies for the development of materials for the needs of electrochemical (bio)sensing.

## 1. Introduction

Vitamins are essential for the proper functioning of the human body. B6 (pyridoxine) is highly influential as a cofactor for enzyme-catalyzed reactions and metabolism of amino acids, partially in the biosynthesis of neurotransmitters [1]. Additionally, it is a paramount vitamin for the formation of red blood cells [2]. Pyridoxine is essential for skin maintenance, and the lack of this vitamin may cause dermatitis [3,4]. Furthermore, administration of B6 may protect an organism from diabetes [5], cardiovascular diseases [6,7] and carcinogenesis [8,9], especially from colon cancer [10,11]. It was recently discovered that pyridoxine serves as an anti-oxidant, performing the function of a radical scavenger [12,13]. But despite this, overconsumption of B6 (500 mg/day and more) may harm the nervous system [7]. The normal concentration of B6 vitamin in human blood is in the range of 5–50 μM [14], and the recommended daily allowance (RDA) is 1.5 mg [15].

The abovementioned points demonstrate that according to its concentration, B6 may change its role from a medicine to a harmful compound. This underlines the necessity for exact and fast determination of the B6 concentration in solutions. Quantitative methods include flow-injection methods with spectrophotometric [16], chemiluminescent [17] and spectrofluorimetric detection [18] as well as chromatography methods [19,20]. The implementation of these methods is time-consuming and needs expensive apparatuses.

Nowadays, electrochemical methods for detection are extremely promising due to their fast response, low cost and low detection limit [21]. There have been a variety of studies devoted to the detection of pyridoxine on glass electrodes modified with carbon nanotubes (CNTs) [22], CuO-CNT composites [23], multi-walled carbon nanotubes [2], metal oxides [24], metal complexes [25] and polymers [26,27].

Rare-earth metals are some of the most promising materials for the modification of carbon electrodes due to their large number of active sites, enhanced conductivity and multi-layer structure [28,29]. The inherence of f-orbitals provides numerous electronic transitions, which enhance electrochemical performance [30]. In the present work, we propose rare-earth borates for the modification of carbon glass electrodes. Facile synthesis of rare-earth borates was performed in one step using a high-temperature annealing procedure [31].

To our knowledge, there has thus far been no comparative study of rare-earth (La, Nd, Dy) borates used for the modification of glass carbon electrodes. In the present study, the physicochemical properties of borates including La, Nd and Dy were characterized using XRD, TEM and FTIR. Using cyclic voltammetry (CV) and electronic impedance spectroscopy (EIS), it was found that DyBO_3_ is the most fitting material for electrode modification. A CG/DyBO_3_ electrode was subjected to vitamin B6 quantitation and showed a low detection limit, high selectivity and reproducibility.

## 2. Materials and Methods

### 2.1. Chemicals and Instrumentation

Boric acid (H_3_BO_3_), acetic acid (CH_3_COOH), phosphoric acid (H_3_PO_4_), lanthanium oxide (La_2_O_3_), neodimium oxide (Nd_2_O_3_), dysprosium oxide (Dy_2_O_3_), potassium chloride (KCl), potassium ferrocyanide (K_4_[Fe(CN)_6_]·3H_2_O) and potassium ferricyanide (K_3_[Fe(CN)_6_]) were purchased from the Chemcraft (Kaliningrad, Russia) company. Glassy carbon spherical powder (CG) was purchased from Sigma-Aldrich (Saint-Louis, MO, USA). The binder (vacuum pump oil) was received from the Edwards company. B6 (pyridoxine) was provided by SternVitamin (Moscow, Russia); all chemicals were analytical grade.

Calcination of rare-earth borates proceeded in a programmable oven provided by Thermopech company (Istra, Russia). The crystalline phase was estimated using a Rigaku (Tokyo, Japan) Ultima IV diffractometer operating with Cu Kα radiation (λ = 0.154 nm). The mean size of anatase crystals was calculated using the Scherrer equation for the (111) plane (LaBO_3_ and NdBO_3_) and for the (002) plane (DyBO_3_). FTIR spectra were registered using a Shimadzu (Kyoto, Japan) IRAffinity1S spectrometer. The TEM study was performed with JEOL (Tokyo, Japan) JEM 2001F. Sample preparation proceeded using dispersion in ethanol, subsequent sonification, deposition on a holey carbon film-coated copper and drying. Electrochemical measurements were performed at an ambient temperature equal to 25 °C on a Corrtest (Wuhan, China) 2350 bipotentiostat. In the redox probe solution, cyclic voltammetry was performed with the scan rates varied from 10 to 300 mV/sec. EIS measurements were performed using a redox probe with current frequencies altered from 50 kHz to 1 Hz, direct current potential equal to 0 V and voltage amplitude = 25 mV. A quantitative study of B6 solutions was performed using the DPV analytical method; the pulse amplitude was 50 mV and the pulse width was 50 ms.

### 2.2. Synthesis of Rare-Earth Borates

**Scheme 1:**
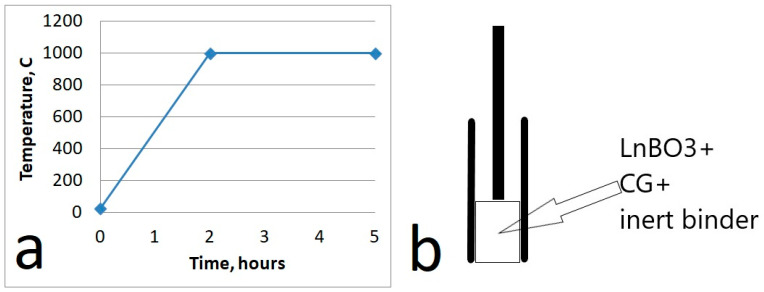
(**a**) Temperature program for the preparation of rare-earth borates. (**b**) Structure of the electrode.

Synthesis was performed according to the following approach [31]. Boric acid was mixed with rare-earth oxide in a molar ratio of 1.5:1 (0.0041:0.0027 moles were taken) and carefully ground in a mortar for 30 min. After this, the mixture was placed into an Al_2_O_3_ crucible with a 15 mL volume. The rate of heating from room temperature up to 1000 °C was 8 °C per minute. Calcination proceeded in a programmable oven provided by the Thermopech company, where heating up to 1000 °C took 2 h with subsequent holding at 1000 °C temperature for 3 h. The materials were cooled in the furnace without any additional cooling program. After cooling, the resulting matter was ground into a powder and weighed without additional washing or drying. The temperature program is presented in Scheme 1a.

### 2.3. Fabrication of Electrodes

The electrode was prepared in the following way: 25 mg of glassy carbon spherical powder (CG) was mixed with 25 mg of rare-earth borate powder and 25 mg of binder (vacuum pump oil) in a mortar. The mixture formed a thick paste, which was tightly packed into a disposable syringe with stainless steel contact. The resulting electrode surface was smooth, and after each measurement, the electrode surface was refreshed by extruding it and removing the outer layer. To prepare the reference CG electrode glassy carbon, the spherical powder was mixed with binder in the absence of rare-earth borate and put into a rigid mold. The structure of the electrode can be seen in Scheme 1b.

### 2.4. Preparation of Solutions

Britton–Robinson buffer solutions ranging from pH 3 to 10 were prepared using a stock mixture of H_3_BO_3_, CH_3_COOH and H_3_PO_4_ each at a concentration of 0.4 M. The desired pH level was achieved by incorporating a 0.2 M solution of NaOH. For the redox solution, a combination of 100 mM KCl and 5 mM ferrocyanide and ferricyanide was used.

To prepare the pyridoxine solutions, a dilution method was used on a stock solution with a concentration of 2 mM. The volume was adjusted to 25 mL, followed by the adding 25 mL of the buffer solution.

## 3. Results

### 3.1. Investigation of Borates Structure

During the synthesis procedure, three materials, namely, LaBO_3_, NdBO_3_ and DyBO_3_, were prepared to modify the glassy carbon electrode.

**Figure 1:**
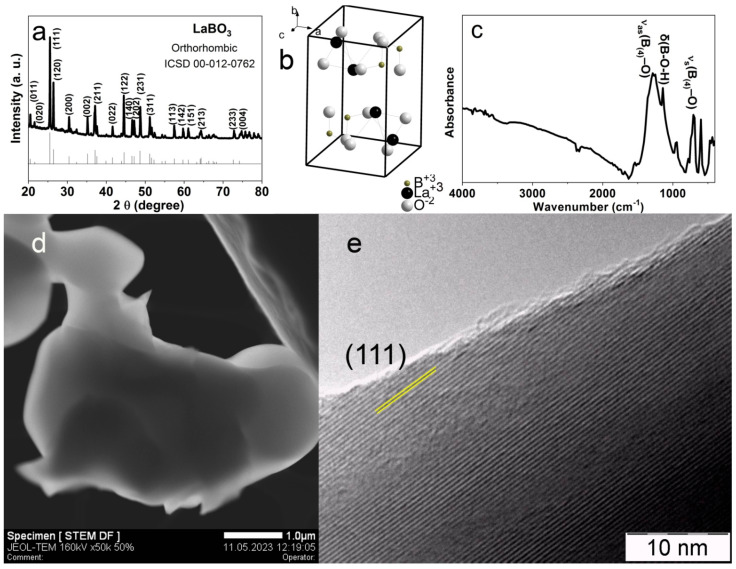
Physicochemical properties of LaBO_3_: (**a**) XRD pattern, (**b**) ball–stick type diagram, (**c**) FTIR absorbance, (**d**) TEM image and (**e**) HRTEM image with the (111) interlayer distance marked in yellow.

The first step of physicochemical studies includes an XRD analysis. The XRD pattern for LaBO_3_ (Figure 1a), showed a striking resemblance to lanthanum borate, with the ICSD (Inorganic Crystal Structure Database) number 00-012-0762. This material exhibited an orthorhombic crystalline phase with a space group Pnma (No. 62). The lattice parameters were determined to be: a = 5.872 angstroms, b = 8.257 angstroms and c = 5.107 angstroms and angles α = 90°, β = 90° and γ = 90° (refer to Table 1) [32]. Figure 1b illustrates a ball–stick diagram, where La is coordinated by four oxygen atoms. Each cell comprises four La atoms, resulting in four stoichiometric units per cell. The FTIR absorbance spectrum shows the (Figure 1c) asymmetric stretching of boron-to-oxygen bands ν_as_(B_(4)_–O) at 1286 cm^−1^, the bending of boron–oxygen–hydrogen δ(B-O-H) at 1138 cm^−1^ and the symmetrical stretching of four-coordinated boron-to-oxygen ν_s_(B_(4)_–O) at 707 cm^−1^ [32]. The morphology of LaBO_3_ is characterized by irregular-shaped particles with sizes in the range of several micrometers (refer to Figure 1d). The high-resolution TEM (Figure 1e) displays the ordered structure of the material, where gaps between the lattice fringes precisely correspond to the interlayer distance of the (111) plain with an XRD peak at 25.5°.

**Figure 2:**
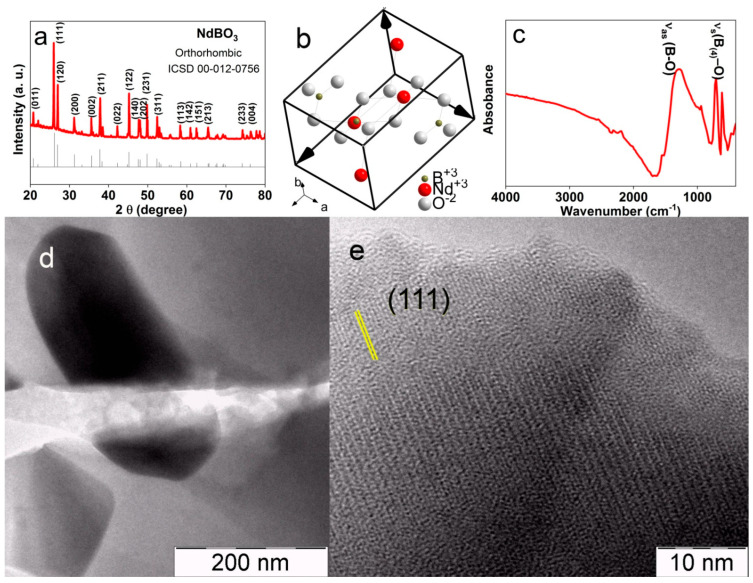
Physicochemical properties of NdBO_3_: (**a**) XRD pattern, (**b**) ball–stick type diagram, (**c**) FTIR absorbance, (**d**) TEM image and (**e**) HRTEM image with the (111) interlayer distance marked in yellow.

Neodymiun borate is classified as an orthorhombic phase, denoted as 00-012-0756 in the ICSD database. Its space group is Pnma (No. 62), as shown in Figure 2a. The lattice parameters of the orthorhombic phase include a = 5.729, b = 8.080 and c = 5.041 angstroms, with all angles measuring 90° (Table 1). The ball–stick type diagram (Figure 2b) demonstrates that each crystalline cell comprises four stoichiometric units. The essential components within the cell structure include BO_3_^3−^ triangles and Nd^3+^, which are coordinated with nine oxygen atoms [33]. The spectrum of infrared absorption (Figure 2c) reveals a broad peak with the center at 1500 cm^−1^, indicating the asymmetric stretching of the B-O bond. Additionally, there is a peak at 900 cm^−1^, which corresponds to symmetric vibrations [34]. The morphology of orthorhombic NdBO_3_ can be described as prism-shaped particles measuring approximately 400 nm in length and 200 nm in width, these dimensions are smaller compared with LaBO_3_ species (Figure 2d). Furthermore, the HRTEM study (Figure 2e) demonstrates the crystalline structure of neodymium borate, with an interlayer distance corresponding to the (111) crystal plane. This specific peak is observed at a position of 25.95 degrees on the XRD graph.

**Figure 3:**
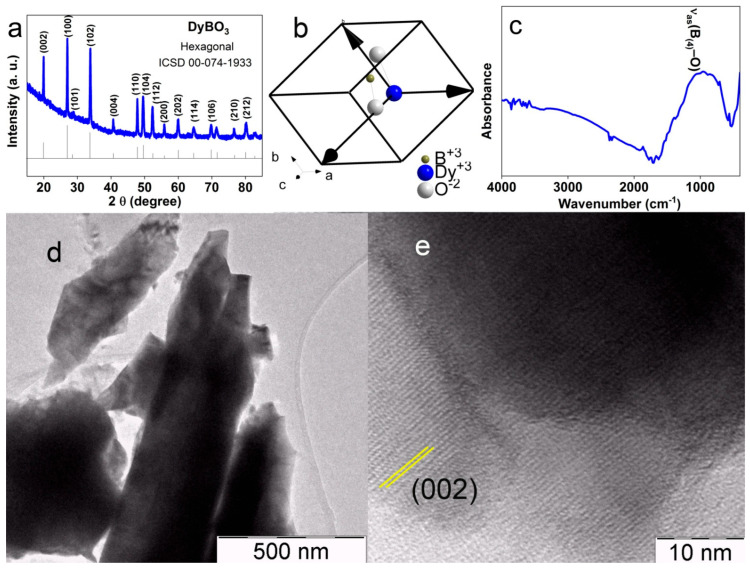
Physicochemical properties of DyBO_3_: (**a**) XRD pattern, (**b**) ball–stick type diagram, (**c**) FTIR absorbance, (**d**) TEM image and (**e**) HRTEM image with the (002) interlayer distance marked in yellow.

Dysprosium borate exhibits a hexagonal crystalline phase, which sets it apart from La and Nd borates. Its corresponding number in the ICSD database is 00-074-1933, with a space group number of P6_3_ mmc (194) (Figure 3a). The cell unit parameters of hexagonal DyBO_3_ are as follows: a = 3.791, b = 3.791 and c = 8.840 angstroms, with angles α = 90°, β = 90° and γ = 120° (Table 1). The ball–stick type diagram shows the vaterite structure of DyBO_3_, wherein the key elements responsible for its formation are tetrahedral polyborate group of B_3_O_9_^9−^, containing BO_4_ groups rather than BO_3_^3−^ anions. On the FTIR spectrum (Figure 3c), a wide band may be seen, which corresponds to the asymmetrical vibration of boron, which is four-coordinated with oxygen [35]. The shape and size of DyBO_3_ particles are given in Figure 3d, revealing rod-like structures measuring micrometers with widths ranging from 300 to 400 nm. The crystalline structure, confirmed by the HRTEM study, indicates that the lattice spacing corresponds to the (200) plane (Figure 3e).

The average crystallite size was calculated using the Scherrer equation:

D = kλ/βcos θ(1)

where D—average crystallite size, nm; k—Scherrer constant equal to 0.95; λ—wavelength = 0.154 nm; β—full-width half maximum (FWHM) and θ—angle of diffraction.

**Figure 4:**
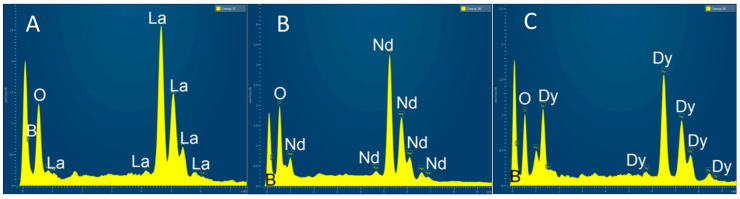
EDX spectra of: (**A**) LaBO_3_, (**B**) NdBO_3_ and (**C**) DyBO_3_.

The calculated values reveal that the average crystallite size along the (111) plane of LaBO_3_ is 59.5 nm, while for the (111) plane of NdBO_3_, it is 60.1 nm. However, it is the (002) plane of DyBO_3_ that stands out with the smallest crystalline size of 43.6 nm. The smaller size suggests that DyBO_3_ possesses a higher specific surface area, as well as the largest number of active sites. As a result, it is expected to exhibit enhanced electrochemical performance in comparison with LaBO_3_ and NdBO_3_. The EDX elemental analysis of borate phases are given in Figure 4.

### 3.2. Conductivity and Electrochemical Activity in Redox Probe

**Figure 5:**
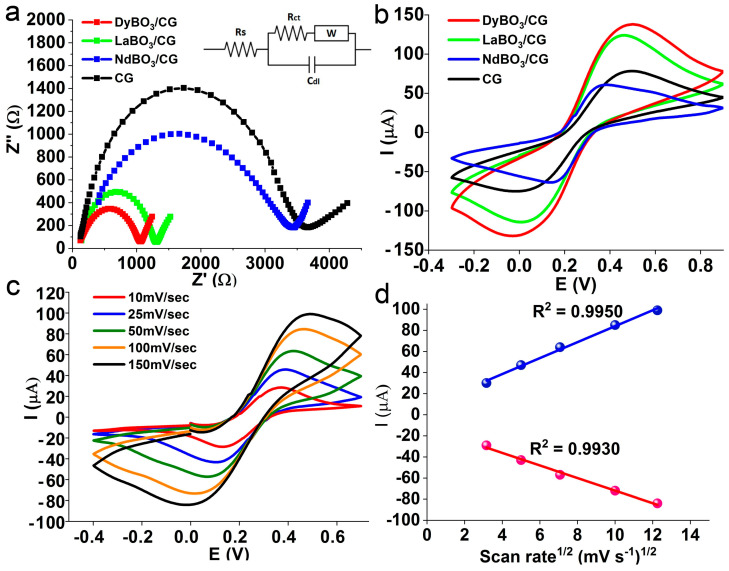
Electrochemical activity of borate-modified electrodes in comparison with pure CG electrode: (**a**) EIS spectra, (**b**) cyclic voltammetry, (**c**) different scan rates for DyBO_3_/CG electrode and (**d**) Randles–Sevcik plot for the DyBO_3_-modified electrode.

Initial and basic electrochemical studies such as cyclic voltammetry and electrochemical impedance spectroscopy (EIS) play a crucial role in determining the most suitable electrode for the quantitative assessment of a target analyte. Cyclic voltammetry allowed for identifying the electrode with the highest signal, while the EIS study showed which electrode had the lowest resistance (impedance). Both studies were performed in a 100 mM KCl solution containing 5 mM of both potassium ferrocyanide K_4_[Fe(CN)_6_] and potassium ferricyanide K_3_[Fe(CN)_6_]. The plot of the impedance spectrum (Figure 5a) shows that all spectra have a half-arc shape with a linear segment at low frequencies, and the Randles equivalent scheme is given in the inset. Within this scheme, R_ct_ is the charge transfer resistance, and it plays a crucial role and corresponds to the size of the semi-arc along the *x*-axis. The lowest value of R_ct_ is inherent to the DyBO_3_-modified electrode, measuring at 930 Ohms. Comparatively, the LaBO_3_-modified electrode exhibits a slightly higher resistance of 1160 Ohms, the NdBO_3_-modified electrode shows a significantly larger resistance value of 3030 Ohms, and the resistance of the unmodified CG electrode is equal to 3540 Ohms. These findings suggest that the DyBO_3_-modified electrode exhibits superior charge mobility and the highest analytical signal compared with the other borates. The smallest crystalline particles of DyBO_3_ contribute to this behavior. The surface of this material includes active sites, which increases the mobility of charges. The Randles–Sevcik equation was used to estimate the electrochemically active surface. At an ambient temperature of 25 °C, it can be expressed as follows [36,37]:

(2) $$i_{p}=2.69\;\times \;10^{5}\;\times \;n^{3/2}\;\times \;\mathrm{AC}\sqrt{Dv}$$

where:

i_p_—Peak current in amps;
n—Number of electrons participating in each redox action;
A—Electrochemical active surface of the electrode in cm^2^;
C—Concentration in mols per mL;
D—Diffusion coefficient in cm^2^/s;
v—Scan rate in V/s.

In order to determine the active surface values of different electrodes, voltammetric studies were performed at a scan rate of 300 mV/s in a 5 mM aqueous solution of ferrocyanide/ferricyanide (Figure 5b). The smallest active surface of 0.0256 cm^2^ corresponds to the NdBO_3_-modified electrode, while the undoped CG electrode has an active surface value equal to 0.0329 cm^2^. The LaBO_3_-doped electrode has a larger surface measuring 0.0516 cm^2^, and the DyBO_3_-doped electrode has a maximum surface area value of 0.0578 cm^2^. The larger the surface, the more active sites are available—this makes the charge transfer easier. From this point of view, the DyBO_3_-modified CG electrode is the most promising—this conclusion is supported by the EIS studies. The influence of the scanning speed on the peak current is shown in Figure 5c,d. As the scan rate increases, the peak current increases because of the reduced diffusion layer near the electrode surface and the subsequent decrease in the concentration gradient. The Randles–Sevcik plot has a linear shape, which indicates that (Figure 5d) the oxidation and reduction processes in solution are controlled by diffusion [38]. It is important to note that the composition of the electrode is not optimized. In order to examine the impact of borate quantity on the electrochemical performance of the electrode, we found that a mass fraction equal to 50% is optimal. A larger percentage of borates in the electrode composition resulted in constant and slight “leakage” of the electrode, which prevented its use. The maximum percentage that ensured a stable and constant signal equaled 50% and, therefore, this amount was used during the test.

### 3.3. Electrochemical Evaluation of Pyridoxine

**Figure 6:**
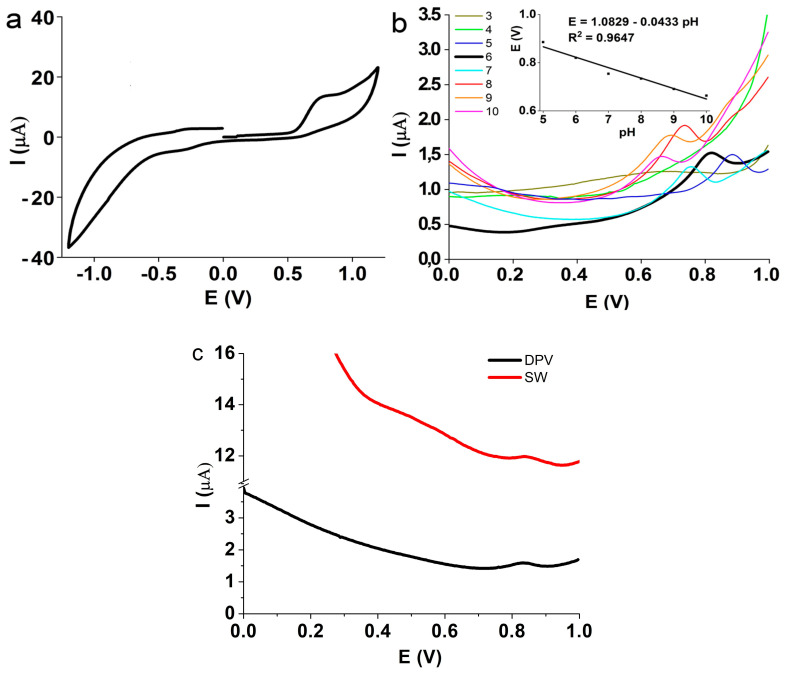
(**a**) Cyclic voltammetry of B6 2 mM solution. (**b**) Effect of pH on the pyridoxine peak signal. Inset: plot of oxidation potential against the pH value. (**c**) Comparison between SW and DPV methods.

The electrochemical determination of vitamin B6 was performed with the most promising DyBO_3_-doped electrode after preliminary CV and EIS tests in redox media. The cyclic voltammogram of the B6 solution demonstrates the absence of the reduction peak of the vitamin (Figure 6a) in the entire tested range. Or, it may be present, but much smaller than the oxidation peak—this indicates that this process is irreversible or quasi-reversible [39]. The evaluation parameters of B6 were optimized before the construction of the calibration graph. One crucial parameter to consider in water solutions is the pH value. To investigate the influence of pH, tests were conducted in Britton–Robinson buffer solutions ranging from pH 3 to pH 10 using the DPV method while maintaining the concentration of vitamin B6 at 100 μM. Figure 6b illustrates the oxidative branch of vitamin B6, where it can be seen that solutions with a pH lower than 5 do not have a peak at all. This happens due to the protonation of B6 in an acidic media, as it can be oxidized only in the deprotonated form. The alteration of the pH to alkaline values expectedly leads to a decrease in the oxidation potential, reaching 0.663 mV at pH 10 [40]. Equation (3) expresses the dependence of B6 oxidation on pH:

E = 1.0829 − 0.0433 pH(3)

The highest current value corresponds to a pH value of 6, which was used for further experiments. This correlates well with the existing literature [39].

**Figure 7:**
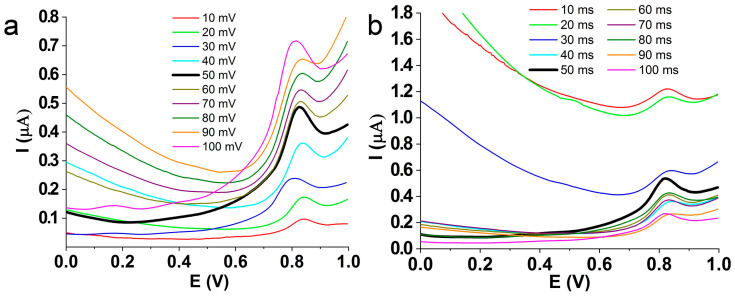
Optimization of DPV parameters: (**a**) pulse amplitude and (**b**) pulse width.

To quantify B6, two powerful electrochemical analytical methods were tested: square wave voltammetry (SWV) and differential pulse voltammetry (DPV). Both methods aim to improve the limit of analyte detection compared with the conventional cyclic voltammetry [41]. Initially, the peak signal obtained using SWV and DPV were compared under the same conditions, namely, a B6 concentration of 100 μM, pH of 6 and amplitude equal to 50 mV (Figure 6c). It was found, that despite the peak current values being almost equal, the SWV exhibited a higher baseline signal, making it less suitable for quantification. Therefore, further measurements were conducted using the DPV method. The main parameters of the DPV method that affect the electrochemical signal are pulse amplitude and duration. The pulse amplitude was varied from 10 to 100 mV while maintaining the pyridoxine concentration equal to 100 μM at pH 6. The maximum peak current was found at 50 mV (see Figure 7a). With this amplitude value, the pulse width was optimized in the range from 10 ms to 100 ms, while the pulse period was equal to 500 ms. The maximum peak current was obtained with a pulse width of 50 ms. All these conditions (pH = 6, pulse amplitude = 50 mV, pulse width = 50 ms) were used for the subsequent study of the calibration graph.

**Figure 8:**
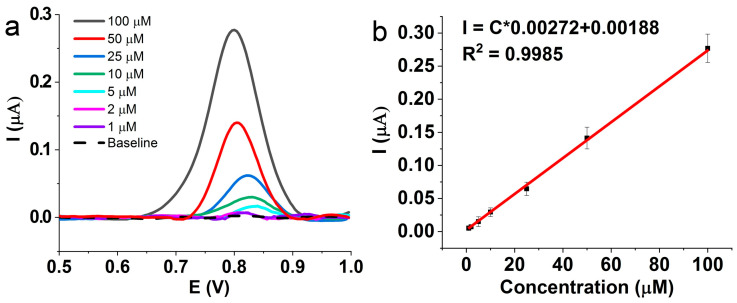
(**a**) Pyridoxine oxidation peaks. (**b**) Calibration graph for pyridoxine oxidation.

Pyridoxine solutions were tested in a concentration range from 1 μM to 100 μM, and the measurements were repeated three times. Figure 8a shows the pyridoxine oxidation signal after subtraction of the baseline, with the maximum current found to be 0.277 μA. The calibration graph (Figure 8b) shows a linear section with a correlation coefficient R^2^ = 0.9985, which can be expressed as the following Equation (4):

I = C × 0.00272 + 0.00188(4)

The limit of detection (LOD), i.e., the lowest analyte concentration that can be detected, and the limit of quantification (LOQ), i.e., the lowest signal value that can be distinguished from the background with reasonable certainty, were calculated using the following formulas [42]:

(5) $$\mathrm{LOD}=3\;\frac{d}{s}$$

(6) $$\mathrm{LOQ}=10\;\frac{d}{s}$$

where:

d—Standard deviation of blank signal (y-intercept of the calibration graph);
s—Slope of the calibration graph.

The following values were found: LOD = 0.051 μM and LOQ = 0.168 μM. The resulting calibration graph range and limits of detection and quantification show the superior electrochemical behavior of the CG/DyBO_3_ electrode for B6 detection. A comparison with other studies dedicated to the detection of pyridoxine is given in Table 2. It may be seen that the CG/DyBO_3_ electrode has superior performance when compared with other types of electrodes that were previously studied for the electrochemical detection of vitamin B6. In Table 2, various electrodes and materials are listed, including a boron-doped diamond electrode, a carbon paste electrode with iron oxide nanoparticles, ZnO nanocrystals anchored on mesoporous TiO_2_, magnetite nanoparticles and multiwalled carbon nanotubes coupled manganese salen. The CG/DyBO_3_ electrode exhibits the lowest LOD values and a wide linear range on the calibration graph, indicating its superiority over the other materials studied.

The LOD value as well as a linear range on the calibration graph are promising for the practical applications of pyridoxine detection. It is worth mentioning that the application of the CG/DyBO_3_ electrode may be limited in certain media that can be harmful to electrode material, for example, non-water media, which can dissolve binding oil and strongly acidic media that can react with DyBO_3_.

A comparison of the electrochemical method for B6 detection with other methods such as spectrophotometry, chemiluminescence, spectrofluorometry and liquid chromatography with a UV detector demonstrates that the electrochemical approach is not inferior to any other method for detection from the point of view of LOD and linear range of the calibration graph. This suggests that the electrochemical method for B6 detection does not need valuable equipment or complicated probe preparation, contrary to other methods (see Table 3).

### 3.4. Reproducibility Experiment

**Figure 9:**
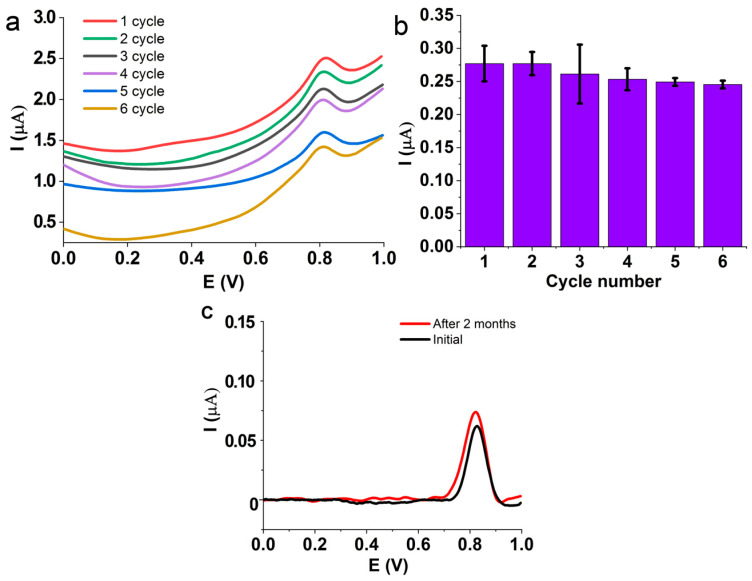
Reproducibility experiment: (**a**) B6 oxidative branch, (**b**) histogram of the electrochemical signal and (**c**) 2-month reproducibility experiment.

The stability of the DyBO_3_/CG electrode was tested in a 100 μM vitamin B6 solution with a pH value of 6. The DPV parameters included an amplitude of 50 mV and a signal width of 50 ms. A set of three electrodes of the same composition was prepared and tested for six measurement cycles. The surface of the electrode was regenerated after each measurement, and the outer layer with a thickness of 1 mm was removed. This experiment showed that the DyBO_3_/CG electrode retained more than 90% of the initial current level in six cycles (refer to Figure 9a,b). The maximum value of the relative standard deviation (RSD) was equal to 6.82%. The electrodes offer promising prospects for practical application due to excellent reproducibility.

The reproducibility of the signal was also studied for a 2-month time span. For this purpose, a single DyBO_3_-modified electrode was studied in the same conditions of 25 uM of the vitamin B6 solution and a pH 6 buffer. Figure 9c shows the results of one measurement followed by another with a time delay of 2 months. It can be seen that the deviation of current does not exceed 15%, even after the long “maturation” time.

### 3.5. Experiment with Interfering Compounds

In practical conditions, electrochemical methods deal with solutions, containing a wide variety of different compounds. A real sensor should distinguish the target analyte even in a multi-component system while maintaining a high level of the analytical signal.

**Figure 10:**
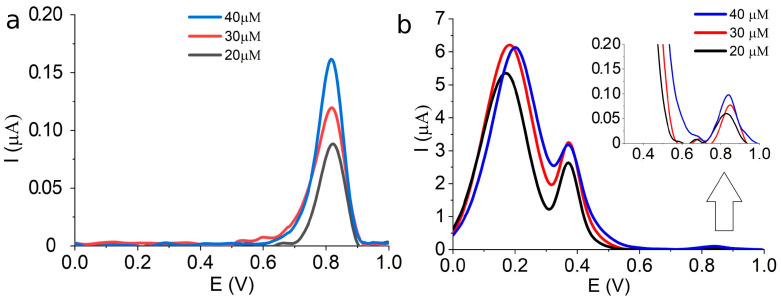
Experiment with interfering compounds: (**a**) oxidation peaks of B6 and (**b**) oxidation of B6 in the presence of interfering compounds.

To study the impact of interfering compounds, the DyBO_3_/CG electrode was tested in artificial urine (AU) mixed with a pH 6 buffer solution. The artificial urine solution was prepared using the dissolution of 200 milligrams of KCl, Na_2_HPO_4_, uric acid, ascorbic acid and glucose, respectively, in 200 mL of distilled water. The result is shown below in Figure 10a, which demonstrates the oxidation peak of B6 in the absence of AU, while Figure 10b shows the electrochemical response of 10 mL AU mixed with 40 mL of the B6 solution. In Figure 10b, the highly intensive peak at 0–0.5 V corresponds to the interfering AU mixture, while B6 oxidation peaks are given in the inset. It can be seen that AU taken in 100-fold excess to B6 cannot restrict its quantitation.

This study established that the DyBO_3_/CG electrode can be applied to the highly selective evaluation of vitamin B6 in concentrated solutions of KCl, Na_2_HPO_4_, uric acid, ascorbic acid and glucose.

In conclusion, it can be said that in the present study, we proposed a stable and confident method for the electrochemical detection of pyridoxine. The detection of B6 with the aid of the DyBO_3_/CG electrode was found to have a low detection limit accompanied by a wide range of calibration graph linearity. The DyBO_3_/CG electrode can be used for at least six cycles without a loss of efficiency, whilst a 100-fold excess of interfering compounds (glucose, ascorbic acid, uric acid, KCl, Na_2_HPO_4_) cannot restrict the evaluation of B6.

## 4. Conclusions

In this study, borates including La, Nd and Dy were synthesized using the solid-state annealing method. The physicochemical properties of the obtained materials were studied using XRD, FTIR and TEM. The crystalline phase, size of crystals, lattice parameters and the dimensions and morphology of the particles were evaluated. It was shown that the prepared materials contain only one borate phase without impurities, and DyBO_3_ material was found to have the smallest size of crystals. The electrochemical properties of modified electrodes were studied in ferrocyanide/ferricyanide redox solution, where the DyBO_3_-modified electrode showed the lowest impedance and the highest peak signal, making it the most promising for B6 detection. The quantification of B6 was performed using the DPV method in Britton–Robinson solution with a pH of 6, and the resulting calibration graph had a linear part from 1 to 100 μM with R^2^ = 0.9985. The reproducibility experiment showed that the electrode retained more than 90% of the initial signal level after six cycles of measurement. And, finally, it was found that the DyBO_3_-modified sensor can be used for the quantification of B6 in the presence of an interfering AU solution, where KCl, Na_2_HPO_4_, uric acid, ascorbic acid and glucose are taken in 100-fold excess in comparison with B6.

## Figures and Tables

**Table 1:** Lattice parameters of MBO_3_ (M = La, Nd and Dy).

Materials	a	b	c	α	β	γ	Volume of Cell, (Å^3^)	Average Crystallite Size (nm)
LaBO_3_	5.872	8.257	5.107	90.00°	90.00°	90.00°	247.613	59.5
NdBO_3_	5.729	8.080	5.041	90.00°	90.00°	90.00°	233.350	60.1
DyBO_3_	3.793	3.793	8.847	90.00°	90.00°	120.00°	110.228	43.6

**Table 2:** Comparison between calibration graph limits and LOD values for various electrodes.

Method	Linear Range	LOD	Reference
DPV	7–47 μM	3.76 μM	[39]
DPV	8.88–1000 μM	9.06 μM	[40]
DPV	2–74 μM	2.5 μM	[43]
SW	200–2000 μM	29.5 μM	[44]
DPV	1–300 μM	0.42 μM	[45]
DPV	1–100 μM	0.051 μM	This work

**Table 3:** Comparison of different methods for pyridoxine detection.

Method	Linear Range	LOD	Reference
Spectrophotometry	5.9–59 μM	0.120 μM	[16]
Chemiluminecsence	0.5–2 μM	0.100 μM	[17]
Spectrofluorimetry	0.6–23.7 μM	0.440 μM	[18]
Liquid chromatography with UV detector	0.012–35.5 μM	0.030 μM	[19]
DPV	1–100 μM	0.051 μM	This work

## Data Availability

This article is a free-access publication.
